# Monitoring of Pesticide Residues in Chili Peppers Using International Pesticide Monitoring Data for Safety Management

**DOI:** 10.3390/toxics12070508

**Published:** 2024-07-15

**Authors:** Minsoo Park, Seo-Hong Kim, Subin Bae, Moo-Hyeog Im

**Affiliations:** 1 Korea Advanced Food Research Institute of Korea Food Industry Association, Uiwang 16001, Republic of Korea; qkralstnrla@naver.com (M.P.); bsub217@naver.com (S.B.); 2 Department of Environmental and Biological Chemistry, Chungbuk National University, Cheongju 28644, Republic of Korea; 3765ksh@hanmail.net; 3 Department of Food Engineering, Daegu University, Gyeongsan 38453, Republic of Korea

**Keywords:** chili pepper, imported food, pesticide residue, pesticide monitoring, safety management

## Abstract

Repeated pesticide residue detection in chili peppers in the Republic of Korea has become a serious health concern. Thus, monitoring domestically grown and imported chili peppers for pesticide residues is of great significance. Here, we investigated pesticide residues detected in imported and domestically grown chili peppers using global pesticide residue monitoring data. Our analysis involved organizing inspection and detection data from different sources. Global pesticide residue monitoring data for chili peppers revealed 139 pesticide types, 43,532 inspections, and 3966 detections (detection rate, 9.11%). Peppers from Mexico were sampled the most (39,927 inspections) and showed the highest number of detected cases (2998 cases). Globally, the top 10 most frequently detected pesticides were clothianidin, imidacloprid, thiamethoxam, chlorpyrifos, thiacloprid, metalaxyl, myclobutanil, azoxystrobin, carbendazim, and cyhalothrin, with detection rates in the range of 10.52–28.66%. Furthermore, domestic chili pepper pesticide residue monitoring revealed 73 pesticide types, 3535 inspections, and 332 detected cases (detection rate, 9.39%), and the top 10 most frequently detected pesticides were chlorfenapyr, tebuconazole, flonicamid, dinotefuran, boscalid, pyraclostrobin, fluxametamide, thiamethoxam, pyridaben, and azoxystrobin, with detection rates in the range of 13.89–32.58%. These findings may serve as fundamental data for safety management related to chili pepper pesticide residues in the Republic of Korea.

## 1. Introduction

Chili peppers (e.g., *Capsicum annuum* L.), which are fruit vegetables and members of the nightshade family (Solanaceae), thrive in tropical climates [1], and their main characteristic feature is their spicy taste, which is primarily due to the presence of capsaicinoids, particularly, capsaicin and dihydrocapsaicin. Furthermore, different chili pepper types have different capsaicinoid contents [2].

In 2020, the production of chili peppers in the Republic of Korea was 60,076 tons, with 57.3% of this amount produced in three provinces, mainly Gyeongsangbuk-do (16,955 tons), Jeollanam-do (9941 tons), and Jeollabuk-do (7573 tons) Provinces [3]. In 2020, the average daily per capita intake of chili peppers in the Republic of Korea was approximately 4.51 g/day. Thus, among the vegetables consumed in this country, chili peppers rank high, at the 17th position. Further, red pepper paste, a common processed form of red pepper, ranks as the second most consumed spicy product in the Republic of Korea (5.51 g/person/day) [4]. Thus, chili peppers, in both their raw and processed forms, are extensively consumed in the Republic of Korea [5]. Despite its substantial domestic production, large amounts of chili pepper are still imported into the Republic of Korea annually. For example, in 2020, chili pepper importation, primarily from China, exceeded domestic production by four times, reaching 246,291 tons [6]. However, there have been health-related concerns regarding the importation of chili pepper owing to the detection of pesticide residues in the imported products. For example, isoprocarb residues, chlorobenzuron and flutriafol residues, tolfenpyrad residues, and chlorfenpyr and pyraclostrobin residues were detected in imported chili peppers in 2020, 2019, 2018, and 2016, respectively [6,7,8,9]. Therefore, considering the high level of chili pepper importation into the Republic of Korea and the identification of these pesticide residues, rigorous inspection and regulation are necessary not only for chili peppers produced domestically, but also for those imported into the country.

To address the abovementioned concerns, the Korean Ministry of Food and Drug Safety (MFDS) monitors pesticide residues in imported foods, aiming to ensure compliance with established maximum residue limits (MRLs). When the detected level of a pesticide residue in imported foods exceeds the MRL, the food product is classified as non-compliant and its importation may be restricted thereafter. However, the detection or non-detection of pesticides in food products depends on whether or not their pesticide residues exceed the limit of quantification (LOQ), which refers to the minimum detectable amount of a given pesticide in a sample. Additionally, in the absence of a defined MRL, a positive list system (PLS), which enforces a uniform standard MRL (0.01 mg/kg), is used to ensure safety [10,11]. Thus, the monitoring of pesticide residues ensures adherence to good agricultural practice (GAP) and may lead to the prevention of consumer exposure to pesticides exceeding the MRL [12]. Food imported into South Korea undergoes four types of inspections, namely, document, on-site, precision, and random sampling inspections, before it is categorized as compliant. Specifically, document inspection involves the evaluation of compliance based on submitted documents, while on-site inspection focuses on assessing the overall attributes of the product, including appearance, taste, and color. Furthermore, precision inspection involves the application of physical, chemical, and microbiological analytical methods and is primarily conducted on food products imported for the first time or those associated with risk information. Finally, random sampling inspection involves the examination of non-targeted foods using physical, chemical, and microbiological methods and is performed to determine compliance with established norms. In 2019, a total of 738,082 foods were imported into the Republic of Korea, and the most frequently employed inspection method for these food products was document inspection (473,259 cases, 64.1%), followed by on-site inspection (125,391 cases, 17.0%), precision inspection (100,532 cases, 13.6%), and random sampling inspection (38,900 cases, 5.3%) [7]. Additionally, pesticide residue monitoring has been ongoing in South Korea since 1968, and legal frameworks for monitoring domestically distributed agricultural products annually are well established. For example, in 2014 and 2017, the Ministry of Food and Drug Safety monitored 517 and 500 agricultural products, respectively, and in 2012, the Institute of Health and Environment of the Metropolitan Government of Seoul monitored pesticide residues in 14,649 agricultural products distributed in Seoul [13]. Owing to the large number of inspections conducted in the Republic of Korea, pesticide violation rates related to imported foods are notably lower than those obtained for other countries. In 2018, for example, pesticide detection based on laboratory and random sampling tests was conducted for a total of 149,755 cases of imported foods, and thus, 1987 violations (2.22%) were identified [14]. Furthermore, in the United States of America (USA), 2956 inspections for the identification of pesticides in imported food in 2018 uncovered 382 violations, implying a violation rate of 12.9%, which is considerably higher than that obtained for the Republic of Korea [15].

Country differences with respect to pesticide application methods as well as varying MRLs and LOQs have been reported. These discrepancies underscore the need to use global pesticide residue monitoring data to develop informed and effective safety management strategies for imported foods. Furthermore, depending on the LOQ, the detection or non-detection of pesticides in food can vary even for the same residue levels. Also, a pesticide residue level deemed acceptable overseas may exceed the MRL in the Republic of Korea, leading to the product being categorized as non-compliant. Therefore, analyzing data on chili pepper residues for different countries offers the possibility of identifying countries where chili pepper pesticide residues are most frequently detected and also enables the comparison of the pesticide residue levels in these imported samples against the MRL in the Republic of Korea. The results of such a comparison can significantly enhance compliance prediction and also serve as a theoretical basis for accurately planning and executing inspections for pesticide detection. Therefore, in this study, we investigated global pesticide residue monitoring data to identify pesticides that are frequently detected in chili peppers. To this end, we collected and organized data on inspection frequency, detection instances, and detected pesticides with the goal of enhancing safety management. The findings of this study may serve as a basis for the safety management of pesticide residues in chili peppers imported into the Republic of Korea.

## 2. Materials and Methods

### Collection of Pesticide Residue Monitoring Data for Chili Peppers

To assess the global status of pesticide residues in chili pepper, we collected and organized monitoring data from the USA, the United Kingdom (UK), Japan, and the European Union (EU). Then, for comparison with the global data, we also collected data for the Republic of Korea.

In the USA, the Food and Drug Administration (FDA) monitors pesticide residues in food products, compiling and publishing annual reports in this regard. Thus, pesticide residue monitoring data for the USA spanning the 2015–2019 period were retrieved from the FDA website [16,17].

In Japan, various institutions, such as the National Institute of Health Sciences (NIHS) and the Local Sanitation Research Institute, work together under the supervision of the Ministry of Health, Labor, and Welfare (MHLW) to monitor pesticide residues in crops. Thus, pesticide residue monitoring data for imported food, including violations and corresponding to the 2016–2020 period, were sourced from the MHLW website [18,19].

In the EU, each member country conducts its own pesticide residue monitoring, complemented by joint monitoring efforts. Relevant authorities then collect and publish the monitoring results, including violation data, which are then made accessible by the Rapid Alert System for Food and Feed (RASFF). For this study, we collected data spanning the 2017–2021 period from the RASFF [20,21,22]. While the EU data generally contain information on the number of samples, inspections, and detections, only detection rate data were available for some pesticides with high detection frequencies. In such instances, the detection rate was converted to the number of detections, and if the number of detections was unclear, the data were presented as a single case.

Furthermore, in the UK, the Health and Safety Executive (HSE) monitors pesticide residue in both domestically produced and imported food products, and the collected data are reviewed and published by the Expert Committee on Pesticide Residues in Food (PRiF). For this study, we collected data corresponding to the 2015–2019 period from annual reports published by the HSE [23,24]. 

For the Republic of Korea, pesticide residue monitoring data for domestic food products for the 2015–2021 period were retrieved from research reports published by the National Institute of Food and Drug Safety Evaluation (NIFDS), available on the MFDS website [25,26,27,28,29,30,31].

The collected data for the different countries were organized by release year, inspection country, country of origin, detected pesticides, number of inspections, number of detections, and residual amount detected. Pesticide names were standardized based on domestic testing methods, with inconsistencies resolved according to domestic standards. Only data for pesticides with detection levels above the limit of quantification (LOQ) were analyzed. For data that did not specify the number of inspections or detections, we assumed that the number of detections was equal to the number of inspections. In cases where the mean, maximum, and minimum residual amounts were provided, the maximum residual amount was used. Furthermore, instances without the specification of the residual amount were denoted as blank.

## 3. Results and Discussion

### 3.1. Monitoring Results for Pesticide Residues in Chili Peppers Worldwide

**Figure 1:**
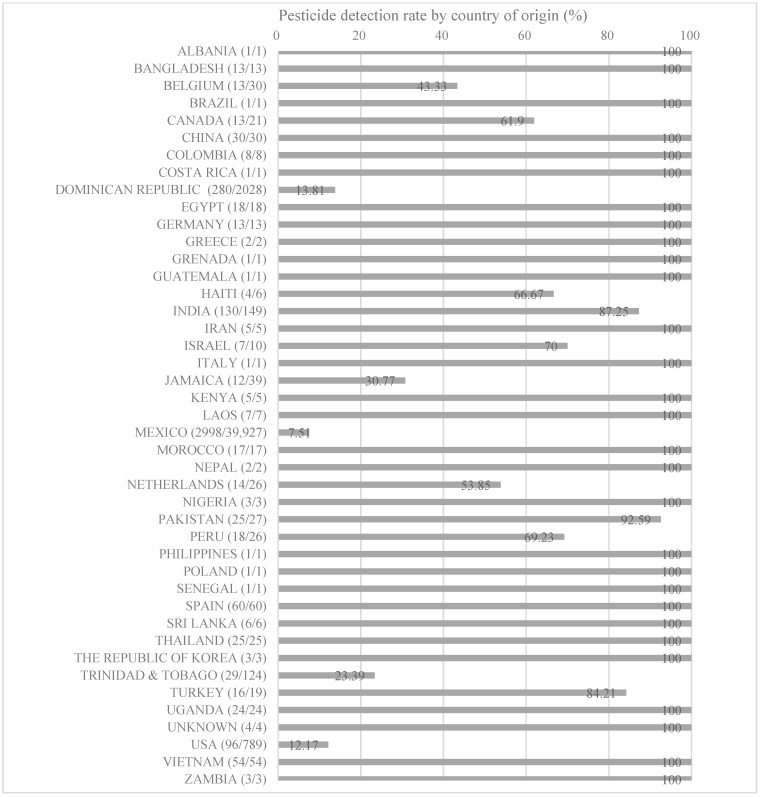
Comparative analysis of pesticide residue inspections and detections in peppers, categorized by country of origin.

A comprehensive analysis of international pesticide residue monitoring data revealed 139 types of pesticides in chili peppers, 43,532 inspections, and 3966 detections, giving a detection rate of 9.11%. Figure 1 shows the overall detection rates for pesticides in food products based on monitoring data from the USA, the UK, the EU, and Japan, categorized by the origin of the food in which the pesticide was detected.

Peppers originating from Mexico were sampled the most (39,927 inspections) and showed the highest number of detected cases (2998). However, the detection rate (7.51%) was significantly lower than that obtained for chili peppers imported from other countries. Conversely, for peppers originating from the Dominican Republic, which were sampled only 2028 times, the number of detected cases was 280, accounting for a detection rate of 13.81%. For peppers from the USA, with 96 detections following 789 inspections, the detection rate was 12.17%. For peppers from India, the detection rate was relatively high, with 103 detections following 149 inspections, giving a detection rate of 69.12%. Furthermore, for chili peppers from Spain and Vietnam, the detection rates were 100%, with 60 and 54 inspections and detections, respectively.

Furthermore, we conducted an analysis to identify pesticides that were frequently detected in chili peppers globally, specifically those with the number of detected cases > 50. These pesticides were evaluated based on the MRL of the Republic of Korea in terms of the number of inspections, number of detections, detection rate, and the maximum residual amount (Table 1). 

The top 10 pesticides with the highest number of detected cases were clothianidin, imidacloprid, thiamethoxam, chlorpyrifos, thiacloprid, metalaxyl, myclobutanil, azoxystrobin, carbendazim, and cyhalothrin, and their detection rates varied from 10.52% to 28.66%. 

Clothianidin exhibited the highest detection rate at 28.66%, showing 280 detections following 977 inspections. This pesticide is effective against various insect species, such as Hemiptera, Coleoptera, Thysanoptera, Lepidoptera, and Diptera. It is also easily absorbed by plants, but it is safe for crops and exhibits low toxicity in mammals, birds, and aquatic species. Additionally, long-term control effects can be achieved even at low doses of clothianidin owing to its high insecticidal activity and chemical stability [32].

The second most detected pesticide was thiamethoxam, which was detected in 277 out of 967 inspections, giving a detection rate of 28.65%. Thiamethoxam is one of the most widely used insecticides, given that it is less toxic to humans but exhibits high activity against pests and insects. However, several countries in Europe placed a ban on its use between 2013 and 2015 owing to concerns regarding its association with the occurrence of honeybee colony collapse disorder [33]. 

Imidacloprid, with 277 detections out of 1014 inspections, showed the third-highest detection rate (27.32%) among the top 10 most frequently detected pesticides. Imidacloprid is a systemic pesticide with a long-life span of up to 190 days, a half-life by photolysis of 39 days, and an indoor half-life of up to 997 days [34].

Chlorpyrifos, the pesticide with the fourth-highest detection rate (20.92%), was detected in 204 out of 975 inspections. This pesticide is a widely used insecticide in agriculture and households, and it is known for its low solubility in water and high soil adsorption coefficient. It also exhibits poor biodegradability and possesses a strong affinity for soil particles, leading to a prolonged persistence of 60–120 days in soil. This characteristic raises environmental pollution concerns, including soil, river, and groundwater contamination [35].

Metalaxyl, which was detected in 123 out of 936 inspections, showed the fifth-highest detection rate among the top 10 pesticides (13.14%). Metalaxyl is a stable compound under a wide range of pH conditions, temperatures, and light intensities. Owing to these characteristics as well as its wide-spectrum activity, it is used on a broad range of crops and in several countries with temperate, subtropical, and tropical climates [36].

Certain pesticides, including metalaxyl, cypermethrin, fipronil, and malathion, exceeded their respective MRLs in chili peppers according to the standards of the Republic of Korea. Furthermore, omethoate, chlorpyrifos, and dimethoate, which lacked a defined MRL, were subjected to a uniform standard of 0.01 mg/kg.

### 3.2. Monitoring Data for Pesticide Residues in Chili Peppers in the Republic of Korea

Domestic pesticide residue monitoring revealed 73 types of pesticides in chili peppers and a total 3535 inspections and 332 detections, accounting for a detection rate of 9.39%. Table 2 presents the pesticides that were detected more than five times based on their respective MRLs in the Republic of Korea, along with information on the number of inspections, detections, detection rates, and maximum residual amounts.

The top 10 pesticides with the highest number of detected cases in the Republic of Korea were chlorfenapyr, tebuconazole, flonicamid, dinotefuran, boscalid, pyraclostrobin, fluxametamide, thiamethoxam, pyridaben, and azoxystrobin, and their detection rates varied in the range of 13.89% to 32.58%. 

The detection rate for chlorfenapyr, with 29 detections out of 89 inspections, was the highest among the top 10 pesticides (32.58%). Chlorfenapyr is an insecticide of the arylpyrrole family, and given that it is a nonpolar compound, it shows limited solubility in water and negligible mobility in soil. Furthermore, its half-life in water is 4.8–7.5 days at pH 4–9 [37].

Dinotefuran, detected in 15 out of 51 inspections, had the second-highest detection rate (29.41%). This neonicotinoid pesticide acts on the nicotinic acetylcholine receptor and, thus, inhibits the expression of the neurotransmitter acetylcholine, causing neurotoxicity. Moreover, it exhibits high permeability. When applied to soil, it is readily absorbed and translocated into crops, and it is primarily used to control thrips and aphids [38]. 

Fluxametamide was detected in 12 out of 51 inspections, yielding a detection rate of 23.53%. Reportedly, fluxametamide, a novel isoxazoline insecticide with a new target site in arthropods, interferes with GABA Cl^−^ and Glu Cl^−^ channels and exhibits exceptional insecticidal activity across various insect species, such as Lepidoptera, Thysanoptera, Coleoptera, and Diptera, with negligible non-target toxicity against pollinators [39].

Further, tebuconazole, detected in 18 out of 79 inspections, was the fourth most frequently detected pesticide domestically (22.78%). Reportedly, tebuconazole is applied to various crops owing to its strong penetration capability, and owing to its ability to effectively control various leaf-patterned diseases and fungi in fruits, it is frequently used as a pesticide [40].

Flonicamid, the fifth most frequently detected pesticide, was detected in 18 out of 89 inspection cases, giving a detection rate of 20.22%. This pesticide belongs to the pyridinecarboxamide group, a novel class of chemical pesticides designed to control aphids resistant to other insecticides. Its insecticidal mechanism primarily involves inducing starvation in pests by inhibiting stylet penetration into plant tissues [41].

Furthermore, a comparison of the maximum residual amounts of the different pesticides with their respective MRLs for the Republic of Korea revealed that none of the pesticides exceeded the established MRLs. Subsequently, comparative analysis involving domestic and global pesticide residue monitoring data (Table 1 and Table 2) revealed that the pesticides detected in both domestic and global monitoring data included chlorfenapyr, fluxametamide, acetamiprid, boscalid, pyraclostrobin, difenoconazole, azoxystrobin, thiamethoxam, clothianidin, carbendazim, and chlorantraniliprole, while those exclusively detected in domestic monitoring data included dinotefuran, fluxametamide, flonicamid, pyridaben, spirotetramat, sulfoxaflor, trifloxystrobin, flubendiamide, tetraconazole, pyflubumide, fluopyram, indoxacarb, and procymidone. Conversely, those detected in global monitoring data and in the Republic of Korea included imidacloprid, chlorpyrifos, metalaxyl, thiacloprid, myclobutanil, methamidophos, cyhalothrin, cypermethrin, omethoate, acephate, dimethodate, fipronil, diflubenzuron, cyfluthrin, bifenthrin, methomyl, and malathion.

Environmental conditions, such as warm and humid climates, are optimal for the proliferation of diverse weeds and the spread of diseases and pests. In countries with such environmental conditions, the use of larger quantities and more pesticide varieties to control pests and diseases are very common [42]. Therefore, variations in pesticide type and regulation, as well as the type of prevalent pests in each country, can be attributed to differences in environmental conditions [43]. Furthermore, pesticides exhibit varying rates of degradation profiles depending on the food type and environmental conditions. The levels of pesticide residues in crops are also affected by the degree of pesticide use, the type of active substance in the pesticide, and the decomposition rate of the pesticide. These factors together contribute to observed differences in pesticide residue levels among countries [44].

### 3.3. Limitations of the Study

This study has some limitations. During the organization of pesticide residue data from different countries, we assumed a detection rate of 100% for cases without inspection or detection counts. This assumption possibly affected the accuracy of the detection rates obtained. Additionally, owing to varying pesticide LOQs across countries, substances categorized as above the LOQ in one country may be categorized as non-detectable in another country with a lower LOQ. This issue possibly led to inconsistencies in the detection results obtained. For example, the U.S. Department of Agriculture (USDA) collects pesticide residue data via the Pesticide Data Program (PDP), which uses lower LOQs than the FDA. These differences possibly result in different detection outcomes for the same residue levels. These limitations raise concerns regarding the feasibility of making valid comparisons when analyzing data collected from different countries.

## 4. Conclusions

In this study, we investigated global pesticide residue monitoring data for chili peppers. Our results revealed 43,532 inspections and 3966 detected cases, implying a detection rate of 9.11%, and globally, the top 10 detected pesticides were clothianidin, imidacloprid, thiamethoxam, chlorpyrifos, thiacloprid, metalaxyl, myclobutanil, azoxystrobin, carbendazim, and cyhalothrin. Conversely, domestic pesticide residue monitoring revealed 3535 inspections and 332 detections, and hence a detection rate of 9.39%, and the top 10 pesticides detected were chlorfenapyr, tebuconazole, flonicamid, dinotefuran, boscalid, pyraclostrobin, fluxametamide, thiamethoxam, pyridaben, and azoxystrobin. Additionally, pesticides detected solely in domestic monitoring included dinotefuran, fluxametamide, flonicamid, pyridaben, spirotetramat, sulfoxaflor, trifloxystrobin, flubendiamide, tetraconazole, pyflubumide, fluopyram, indoxacarb, and procymidone, while those detected solely in global monitoring included imidacloprid, chlorpyrifos, metalaxyl, thiacloprid, myclobutanil, methamidophos, cyhalothrin, cypermethrin, omethoate, acephate, dimethodate, fipronil, diflubenzuron, cyfluthrin, bifenthrin, methomyl, and malathion. Based on our findings, it is possible to identify the specific origin of chili pepper based on the pesticides detected. Our results also indicated that peppers originating from Mexico, the Dominican Republic, the USA, India, Spain, and Vietnam were extensively sampled or showed high pesticide detection rates. Therefore, conducting thorough inspections of peppers imported from these countries could be a viable approach for the safety management of pesticide residues. Moreover, rigorous inspection protocols can be established by identifying prevalent pesticides in both imported and domestically grown peppers to ensure enhanced safety measures. Additionally, our results showed high occurrences and detection rates for pesticides such as clothianidin, thiamethoxam, imidacloprid, and chlorpyrifos overseas. Similarly, domestic distribution monitoring data showed high occurrences and detection rates for chlorfenapyr, tebuconazole, flonicamid, and dinotefuran. Considering these pesticides as targets for thorough inspection during the importation or domestic distribution of chili peppers might be a plausible safety management strategy. It is also recommended to include pesticides, such as imidacloprid, chlorpyrifos, metalaxyl, thiacloprid, and myclobutanil, which are frequently detected overseas, but not domestically, in inspection targets during the inspection of imported products. Such measures may ensure comprehensive inspection given that pesticides that are not frequently detected domestically may not currently be designated for thorough inspection during importation. Additionally, it is crucial to assess whether sufficient inspection measures are in place for pesticides that are frequently detected domestically; otherwise, comprehensive inspection may be necessary. Currently, 113 types of pesticides with a high number of violations are being sampled in the Republic of Korea regardless of the type of food. However, myclobutanil and acephate, detected in peppers overseas, and flonicamid, fluxametamide, spirotetramat, sulfoxaflor, tetraconazole, fluopyram, and pyflubumide, detected in peppers grown domestically, are currently not among the designated 113 pesticides subject to inspection in the Republic of Korea. Therefore, adding these pesticides for thorough inspection of peppers may enhance safety management.

In future studies, pesticides with high violation or detection rates for different food types can be investigated, and then the pesticides to be sampled can be designated for each food type. Furthermore, with such comprehensive studies, it is expected that pesticides with high detection rates will be identified, thereby enhancing thorough pesticide safety management and decreasing inspection costs.

## Figures and Tables

**Table 1:** Global trends in pesticide residue detection in chili peppers (2015–2021).

No.	Detected Pesticide	Number of Sampled Cases	Number of Detected Cases	Detection Rate (%)	Residue Concentration (Max, mg/kg)	MRL ^a^ (mg/kg)
1	Clothianidin	977	280	28.66	0.10	2.0
2	Thiamethoxam	967	277	28.65	0.01	1.0
3	Imidacloprid	1014	277	27.32	0.02	1.0
4	Chlorpyrifos	975	204	20.92	0.09 ^c^	-
5	Metalaxyl	936	123	13.14	1.83 ^b^	1.0
6	Thiacloprid	973	125	12.85	0.02	1.0
7	Myclobutanil	903	114	12.62	0.03	1.0
8	Azoxystrobin	933	111	11.90	0.02	2.0
9	Methamidophos	849	97	11.43	0.06	1.0
10	Cyhalothrin	932	98	10.52	0.44	0.5
11	Boscalid	900	93	10.33	0.03	3.0
12	Acetamiprid	946	83	8.77	0.63	2.0
13	Pyraclostrobin	946	82	8.67	0.23	1.0
14	Chlorantraniliprole	837	72	8.60	0.01	1.0
15	Cypermethrin	908	78	8.59	0.79 ^b^	0.5
16	Carbendazim	1277	107	8.38	0.45	5.0
17	Omethoate	858	71	8.28	0.07 ^c^	-
18	Chlorfenapyr	879	72	8.19	0.04	1.0
19	Acephate	882	71	8.05	0.06	3.0
20	Dimethoate	860	69	8.02	0.01 ^c^	-
21	Fipronil	807	63	7.81	0.22 ^b^	0.05
22	Diflubenzuron	812	63	7.76	0.22	2.0
23	Cyfluthrin	766	59	7.70	0.10	1.0
24	Tebuconazole	897	69	7.69	1.26	3.0
25	Bifenthrin	857	65	7.58	0.01	1.0
26	Difenoconazole	848	64	7.55	0.03	1.0
27	Methomyl	905	66	7.29	0.69	5.0
28	Malathion	762	52	6.82	0.40 ^b^	0.1

^a^ Korean MRLs as of January 2024; ^b^ Residues exceeding Korean MRLs; ^c^ Residues without set MRLs.

**Table 2:** Domestic trends in pesticide residue detection in chili peppers (2015–2021).

No.	Detected Pesticide	Number of Inspections	Number of Detections	Detection Rate (%)	Residue Concentration (Max, mg/kg)	MRL ^a^ (mg/kg)
1	Chlorfenapyr	89	29	32.58	0.75	1.0
2	Tebuconazole	79	18	22.78	2.68	3.0
3	Flonicamid	89	18	20.22	0.36	2.0
4	Dinotefuran	51	15	29.41	0.24	2.0
5	Boscalid	89	14	15.73	0.09	3.0
6	Pyraclostrobin	89	14	15.73	0.38	1.0
7	Fluxametamide	51	12	23.53	0.21	1.0
8	Thiamethoxam	89	11	12.36	0.38	1.0
9	Pyridaben	51	10	19.61	0.22	5.0
10	Azoxystrobin	72	10	13.89	0.53	2.0
11	Acetamiprid	51	9	17.65	0.15	2.0
12	Spirotetramat	51	9	17.65	1.75	2.0
13	Trifloxystrobin	79	9	11.39	0.50	2.0
14	Sulfoxaflor	51	8	15.69	0.07	0.5
15	Tetraconazole	79	8	10.13	0.13	1.0
16	Clothianidin	62	7	11.29	0.34	2.0
17	Procymidone	89	7	7.87	0.76	5.0
18	Difenoconazole	41	6	14.63	0.28	1.0
19	Fluopyram	62	6	9.68	0.68	3.0
20	Indoxacarb	72	6	8.33	0.08	1.0
21	Flubendiamide	48	5	10.42	0.40	1.0
22	Carbendazim	51	5	9.80	0.39	5.0
23	Pyflubumide	51	5	9.80	0.17	1.0
24	Chlorantraniliprole	72	5	6.94	0.06	1.0

^a^ Korean MRLs as of January 2024.

## Data Availability

The data presented in this study are available on request from the corresponding author owing to legal restrictions.
